# Fellowships, Grants, & Awards

**Published:** 2005-12

## Ecology and Oceanography of Harmful Algal Blooms

The U.S. Environmental Protection Agency (EPA), as part of its Science to Achieve Results (STAR) program, and its interagency partners, the National Oceanographic and Atmospheric Administration (NOAA), the National Aeronautic and Space Administration (NASA), and the Office of Naval Research (ONR), are seeking applications proposing targeted research projects of up to 3 years duration and, depending on appropriations, multidisciplinary regional studies for 3–5 years for the Ecology and Oceanography of Harmful Algal Blooms (ECOHAB) program. This program supports research on algal species whose populations may cause or result in deleterious effects on ecosystems and human health. Studies of the causes of such blooms, their detection, effects, mitigation, and control in U.S. coastal waters (including estuaries and Great Lakes) are solicited.

Harmful algal blooms (HABs) are caused by a diverse group of organisms, including toxic and noxious phytoplankton, some protists, cyanobacteria, benthic algae, and macroalgae. While some HABs occur naturally, others may be stimulated by human activities. Blooms can extend over large geographic areas, be composed of more than one harmful or toxic species, and cause significant impacts on fisheries, recreation, human health, and the ecology of both marine and fresh water bodies. HABs are now a recurrent and serious problem in many areas of the United States, and evidence suggests that the frequency and distribution of HABs is also increasing globally, impacting many countries that have commercial and recreational activities in the coastal ocean.

HAB impacts on public health and local/regional economies are also dramatic and increasing. In a recent study, average annual economic losses in the United States from HABs were approximated at $49 million, with costs attributable to maintenance of toxin monitoring programs; closures of shellfish beds; marine mammal stranding networks; collapse of some fisheries; mortality of fish, shellfish, turtles, birds, and mammals; disruptions in tourism; threats to public and coastal resource health; publication of watershed, health, and seafood advisories; and medical treatments (Anderson et al. 2000, available at http://www.whoi.edu/redtide/pertinentinfo/Economics_report.pdf). Despite greater public awareness and advisories of bloom events, human illnesses and even fatalities continue to be reported. Additionally, some toxins may cause only a few documented illnesses but result in serious public reaction and temporary aversion to local seafood products and activities (e.g., $46 million in lost revenue from; the 1997 Maryland fish health/*Pfiesteria* events; Anderson et al. 2000). These deleterious impacts have increased public awareness and demand for intervention to reduce or eliminate bloom impacts on coastal resources, local economies, and threats to public health.

Over the course of the last decade, numerous national and agency reports have described the magnitude of the HAB problem and outlined research plans to systematically address the issue. The ECOHAB Program was initiated a decade ago as an interagency, scientific program designed to increase the understanding of the fundamental processes underlying the impacts and population dynamics of HABs (ECOHAB 1995). Three major research themes encompassing the priorities of issues of national importance on the HAB phenomenon were identified: 1) organisms, with a goal towards determining the physiological, biochemical, and behavioral features that influence bloom dynamics; 2) environmental regulation, with a goal toward determining and parameterizing the factors that govern the initiation, growth, and maintenance of these blooms; and 3) food web and community interactions, with a goal toward determining the extent to which food webs and trophic structure affect and are affected by the dynamics of HABs. Information in these areas, in turn, supported a critical goal of the ECOHAB program, the development of reliable models to forecast bloom development, persistence, and toxicity. Since 1997, the ECOHAB Program has sponsored nearly 100 projects with topics ranging from molecular aspects of HAB detection to large-scale, multidisciplinary regional studies of bloom formation, maintenance, and dissipation. Projects cover a wide spatial spectrum along the U.S. coastline and its territories. ECOHAB-sponsored projects also address the detection, prevention, control, and mitigation of HABs and their impacts, as well as economic assessments of these recurring events. Project summaries may be viewed at http://www.whoi.edu/science/B/redtide/nationplan/ecohabprojectsummaries.html

Although several research efforts have been completed or are underway, the understanding of the biological, physical, and chemical processes that regulate HABs remains limited. Toxic blooms can impact virtually all compartments of marine food webs, resulting in adverse effects on metabolism, viability, growth, fecundity, and recruitment of marine organisms. HAB-produced toxins can have immediate, acute impacts on marine populations, including marine mammals, birds, and several protected species. Little is known about the effects of chronic, low-level exposure. Dramatic shifts in ecosystem structure can result from plankton blooms and macroalgal overgrowth in benthic systems. In this context, present knowledge is inadequate to define the scale and complexity of many HAB phenomena.

As a result, an additional focus on the early detection of bloom species, the environmental conditions supporting blooms, and the toxins associated with some HAB species is needed. Further, while there is increasing emphasis on manipulating coastal waters to prevent or control HABs in other nations, it is practically absent from U.S coastal management strategies. Finally, there needs to be greater emphasis on ensuring that coastal managers and the public are provided the most current information available in a manner that will maximize its usefulness in mitigating HAB impacts. This would include use of observing systems and models in the development of HAB forecasts.

The solicitation closing date is January 10, 2006. Funding is contingent upon receipt of fiscal years 2006–2010 federal appropriations. It is anticipated that a total of approximately $7–10 million will be awarded, depending on the availability of funds. The agency partners anticipate awarding approximately 15–20 funding agreements under this solicitation, including two regional projects. Awards for targeted studies are typically on the order of $150,000 per year, total costs, for up to 3 years. Multi-investigator and multi-institutional applications may include correspondingly higher budgets and longer project periods, but may not exceed a 5-year project period.

You may submit either a paper application or an electronic application (but not both) for this solicitation. The necessary forms for submitting a paper application will be found on the National Center for Environmental Research website, http://es.epa.gov/ncer/rfa/forms/. To apply electronically you must use the application package available at https://apply.grants.gov/forms_apps_idx.html (see "Submission Instructions for Electronic Applications") plus some additional forms from http://es.epa.gov/ncer/rfa/forms/

Contact: Bronda Harrison, 202-343-9777, e-mail: harrison.bronda@epa.gov

## Implications of Tropospheric Air Pollution for Surface UV Exposures

The U.S. Environmental Protection Agency (EPA), as part of its Science to Achieve Results (STAR) program, is seeking applications proposing research to better understand the effect of tropospheric pollution (ozone and particulate matter) on surface ultraviolet radiation levels.

Exposure to ultraviolet radiation (UV) has significant health and ecosystem impacts. Under current conditions, one in five Americans will develop skin cancer in their lifetime and one American dies every hour from this devastating disease. Changes in air quality affect UV exposures in ways that we do not fully understand. The goal of this solicitation is to enable research to better understand how changes in tropospheric ozone and particulate matter will alter surface UV exposures.

Several different U.S. government agencies operate UV monitoring programs that employ instrumentation falling into three distinctly different categories: broad-band sensors of radiation intensity within a spectral region between two specified levels, narrow-band instruments measuring radiation in several well-defined wavelength intervals, and spectral devices providing detailed information on radiation intensity as a function of wavelength. Details about the various UV monitoring networks can be found via the web at: http://www.arl.noaa.gov/research/programs/uv.html

Between 1996 and 2004, EPA, in conjunction with the National Park Service, operated a 21-site monitoring network with a goal of characterizing UV exposures and trends in a range of ecoregions across the United States. This 21-site network used Brewer Mark IV single-monochromator spectrophotometers, which measure UV radiation levels reaching the Earth’s surface at 0.5 nm increments over the wavelength range 286.3–363 nm, providing information on UV-B and UV-A exposures. The data from this network are available via the web at http://www.epa.gov/uvnet/. A review of the data collected by this network was recently completed and is available in draft form at http://www.geecresearch.com/EPAUV.htm

EPA is currently working with the National Oceanographic and Atmospheric Administration’s (NOAA’s) Central UV Calibration Facility (CUCF) to redeploy its Brewer instruments at six sites, some of which are part of networks operated by NOAA and the U.S. Department of Agriculture. The new network will include a triad of Brewers at CUCF’s Table Mountain Test Facility, where the Brewers will be compared to NOAA’s reference spectro-radiometer. The new network configuration is intended to help characterize the performance of the Brewers and to collect information relevant to quantifying the effects of tropospheric ozone and fine particles on surface UV levels and radiative forcing.

The solicitation closing date is January 18, 2006. It is anticipated that a total of approximately $600,000 will be awarded under this announcement, depending on the availability of funds. The EPA anticipates funding approximately two grants under this RFA. The projected award per grant is $100,000 per year total costs, for up to 3 years. Requests for amounts in excess of a total of $300,000, including direct and indirect costs, will not be considered. The total project period for an application submitted in response to this RFA may not exceed 3 years. The EPA reserves the right to reject all applications and make no awards under this RFA. The EPA reserves the right to make additional awards under this RFA if additional funding becomes available. Any additional selections for awards will be made no later than 4 months after the original selection decisions.

You may submit either a paper application or an electronic application (but not both) for this announcement. Instructions for both forms of submission follow. For paper applications, forms can be found on the NCER web site: http://es.epa.gov/ncer/rfa/forms/. For electronic applications, use the application package available at https://apply.grants.gov/forms_apps_idx.html (see “Submission Instructions for Electronic Applications”).

Contact: Bronda Harrison, 202-343-9777; e-mail: harrison.bronda@epa.gov

## Interdisciplinary Partnerships in Environmental Health Sciences

The mission of the National Institute of Environmental Health Sciences (NIEHS) is to promote research that will ultimately reduce the burden of human illness and dysfunction from environmental causes. The research supported by the NIEHS addresses this mission through a diverse grants portfolio consisting of basic *in vitro* and animal research, population-based studies, and a limited number of patient-oriented studies that focus on the understanding, detection, prevention, and intervention of environmentally related disease and disease processes. Recent technological advances and a growing appreciation that environmental factors contribute to most complex diseases provide unprecedented opportunities for developing new research paradigms that bring together interdisciplinary teams of scientists to move basic environmental health sciences research into clinical and public health practice.

The objective of this initiative is to foster scientific collaboration between clinical and basic investigators to accelerate the application of basic research results into the clinical setting to improve human health in those areas where environmental factors are known or expected to influence the development or progression of human disease. Scientific knowledge achieved through this research program is expected to move the field of environmental health sciences into new directions and approaches for the identification, treatment, and prevention of environmentally related diseases or disorders. Through this initiative, the NIEHS will support both the development of new collaborations between researchers with basic and clinical expertise and the continued efforts of existing collaborations. Both activities must directly support the integration of clinical and basic science research.

For the purpose of this solicitation, clinical research is defined as: 1) patient-oriented clinical research conducted with human subjects, or research on the causes and consequences of disease in human populations involving material of human origin (such as tissue or specimens) and for which an investigator or colleague directly interacts with human subjects in an outpatient or inpatient setting to clarify a problem in human physiology, pathophysiology, or disease; 2) development of new clinically based technologies, therapeutic interventions, or clinical trials; 3) epidemiologic and behavioral studies in humans; such studies are appropriate in cases where the primary focus of the study is on a specific disease or disorder and the clinical investigator is an essential part of the planning, conduct, and analysis of the study.

Basic science research is defined as mechanistic research using experimental approaches and may include use of cell lines, *in vitro* or *in vivo* models. Basic research may include the development of new tools to expand the capacity of clinically oriented research.

Applications to this solicitation may be either exploratory in nature, laying the foundation of long-term collaborations or attaining proof of principle for an innovative collaborative approach, or they may be continued development of established collaborations. In either instance, applications must focus on a specific human disease or disorder where there is evidence or a strong rationale for the involvement of environmental factors in its etiology or phenotypic expression and must involve research at both the basic and clinical levels. Suggested topics and example research projects include, but are not limited to, first, integration of basic mechanism-driven and clinical patient-oriented research to gain new insights into the role of environmental factors in complex human diseases: 1) integration of patient phenotype data with high data content techniques such as transcriptomics, proteomics, and metabolomics to investigate the mechanisms by which exposures lead to disease; 2) comparison of animal/model organism and human responses to toxicants to identify biological alterations contributing to the disease etiology; 3) examination of the impact of current and/or prior environmental exposures on the progression, treatment, and survival of patients with existing disease.

Second are collaborations between basic, patient-oriented*,* and epidemiologic researchers to identify and validate biomarkers and apply them to the development or progression of human diseases: 1) application of omics technologies and modeling to existing populations to identify predictive marker profiles for known exposures and the genesis of disease; 2) use of mechanistic response data obtained from the conduct of basic science experimentation to identify putative predictive markers of exposure and response and their validation in existing cohorts; 3) coordination of engineering and clinical expertise to develop systems that integrate exposure with individual biological response or phenotypic change; 4) innovative approaches to the identification of the determinants of individual susceptibility and the interaction between genes and environmental stressors in human disease.

Third is use of environmental stressors as a probe(s) to identify phenotypic variation in humans and animal models of disease to inform genetic analyses of disease susceptibility: 1) conduct of genome-wide association studies to study gene, gene–gene and gene–environment interactions in well characterized cases of environmentally induced disease; 2) identification and assessment of the functional relevance of SNPs and haplotypes associated with environmentally induced disease and the mechanistic consequences of those variations at the molecular and cellular levels.

Fourth are interdisciplinary approaches to the development of intervention and prevention strategies to alter the progression of environmentally induced human disease: 1) identification of novel compounds or engineering of biocompatible materials that protect against, inhibit, or reverse toxicant actions and their validation in disease relevant settings; 2) development of mechanistically derived prevention strategies and application of them in populations with known genetic susceptibility to environmental stressors.

Fifth is collaborative development or refinement and application of model systems that faithfully replicate human disease condition or species comparisons that can be used to understand environmentally induced human disease processes. NIEHS encourages applicants to utilize existing biological and or other resources to address the topics described above where applicable, such as: 1) new analyses of data from completed studies; 2) assay of archived biological samples from completed and ongoing studies; 3) collection of new data and samples from ongoing or completed studies; 4) analysis of public and other accessible databases.

Proposals addressing perturbation of biological processes in the absence of extension to human disease will be considered nonresponsive in the context of this solicitation.

This funding opportunity will use the NIH Exploratory/Developmental Grant (R21) and Research Project Grant (R01) award mechanisms. As an applicant, you will be solely responsible for planning, directing, and executing the proposed project.

This funding opportunity uses just-in-time concepts. It also uses the modular as well as the non-modular budget formats (see http://grants.nih.gov/grants/funding/modular/modular.htm). Specifically, if you are submitting an application with direct costs in each year of $250,000 or less, use the modular budget format described in the PHS 398 application instructions. Otherwise follow the instructions for nonmodular research grant applications.

The PHS 398 application instructions are available at http://grants.nih.gov/grants/funding/phs398/phs398.html in an interactive format. Applicants must use the currently approved version of the PHS 398. For further assistance, contact GrantsInfo at 301-435-0714 (telecommunications for the hearing impaired: TTY 301-451-0088) or by e-mail: GrantsInfo@nih.gov

Applications must be prepared using the most current PHS 398 research grant application instructions and forms. Applications must have a D&B Data Universal Numbering System (DUNS) number as the universal identifier when applying for Federal grants or cooperative agreements. The D&B number can be obtained by calling 866-705-5711 or through the web site at http://www.dnb.com/us/. The D&B number should be entered on line 11 of the face page of the PHS 398 form.

The letters of intent receipt dates for this PAR are December 11, 2005, 2006, 2007, with the application receipt dates January 11, 2006, 2007, 2008. The complete version of this PA is available at http://grants/guide/pa-files/PAR-05-168

Contact: Cindy Lawler, Cellular, Organs and Systems Pathobiology Branch, Division of Extramural Research and Training, National Institute of Environmental Health Sciences, P.O. Box 12233, MD EC-23, Research Triangle Park, NC 27709 USA, 919-316-4671, fax: 919-541-5064, e-mail: lawler@niehs.nih.gov; Kimberly Gray, Susceptibility and Public Health Branch, Division of Extramural Research and Training, National Institute of Environmental Health Sciences, P.O. Box 12233, MD EC-21, Research Triangle Park, NC 27709 USA, 919-541-0293, fax: 919- 316-4606, e-mail: gray6@niehs.nih.gov. Reference PAR-05-168

